# Risk factors for delayed presentation and referral of symptomatic cancer: evidence for common cancers

**DOI:** 10.1038/sj.bjc.6605398

**Published:** 2009-12-03

**Authors:** U Macleod, E D Mitchell, C Burgess, S Macdonald, A J Ramirez

**Affiliations:** 1 General Practice and Primary Care, Division of Community Based Sciences, Faculty of Medicine, University of Glasgow, 1 Horselethill Road, Glasgow G12 9LX, UK; 2 Division of Clinical and Population Sciences and Education, Tayside Centre for General Practice, University of Dundee, Mackenzie Building, Kirsty Semple Way, Dundee, UK; 3 Kings College London, Cancer Research UK Promoting Early Presentation Group, Institute of Psychiatry, St Thomas’ Hospital, London, UK

**Keywords:** delay, diagnosis, systematic review

## Abstract

**Background:**

It has been suggested that the known poorer survival from cancer in the United Kingdom, compared with other European countries, can be attributed to more advanced cancer stage at presentation. There is, therefore, a need to understand the diagnostic process, and to ascertain the risk factors for increased time to presentation.

**Methods:**

We report the results from two worldwide systematic reviews of the literature on patient-mediated and practitioner-mediated delays, identifying the factors that may influence these.

**Results:**

Across cancer sites, non-recognition of symptom seriousness is the main patient-mediated factor resulting in increased time to presentation. There is strong evidence of an association between older age and patient delay for breast cancer, between lower socio-economic status and delay for upper gastrointestinal and urological cancers and between lower education level and delay for breast and colorectal cancers. Fear of cancer is a contributor to delayed presentation, while sanctioning of help seeking by others can be a powerful mediator of reduced time to presentation. For practitioner delay, ‘misdiagnosis’ occurring either through treating patients symptomatically or relating symptoms to a health problem other than cancer, was an important theme across cancer sites. For some cancers, this could also be linked to inadequate patient examination, use of inappropriate tests or failing to follow-up negative or inconclusive test results.

**Conclusion:**

Having sought help for potential cancer symptoms, it is therefore important that practitioners recognise these symptoms, and examine, investigate and refer appropriately.

The possible influence of delays in diagnosis on survival and the risk factors for delay in patients with cancer have been the subject of considerable interest and controversy for many years. Survival from cancer in the United Kingdom is poorer than that of other European countries, and it has been suggested that this can be attributed to more advanced disease stage at presentation (Cancer Research UK, 2009). Consequently, there is a need to understand the diagnostic process, and to ascertain risk factors related to increased time to presentation and treatment. This is particularly relevant in the context of primary care, where many patients present with symptoms that may be indicative of cancer, but where the outcome of the diagnostic process is the exclusion of cancer in most cases. Furthermore, in countries with strong primary healthcare systems, such as the United Kingdom, this is typically the first point of contact for the majority of patients, making early recognition of cancer symptoms even more pertinent.

Minimising time to diagnosis of cancer is dependent on patients presenting with potential cancer symptoms and on primary care practitioners responding appropriately to those symptoms, either by arranging further investigation and/or referring for specialist input. Delay can occur at three phases during the diagnostic process: first, in the interval between the patient first noticing a symptom and first consulting a doctor (often referred to as patient delay), second, between first consultation and referral by a practitioner (doctor or practitioner delay) and finally, between referral and diagnosis (hospital or system delay) (Nichols *et al*, 1981).

In addition to outlining the various points during which extraneous factors might adversely influence time to presentation and referral, considering delay in these phases enables identification of areas where interventions designed to reduce delay might be targeted. However, before such interventions can be developed, we need to better understand the factors that might lengthen or shorten the time taken by patients to seek help for symptoms and gain greater insights into how practitioners (usually in primary care) respond to these symptoms. This topic is not easily amenable to study by randomised trial, and as such, interpreting data available from observational studies is essential to increasing our understanding of the risk factors associated with delay and to facilitating development of effective new strategies to reduce the time involved. If longer delays do impact on survival, as has been shown for breast cancer (Richards *et al*, 1999), such strategies could save a significant number of lives.

## Materials and methods

We report results from two systematic reviews of the world literature. The first relates specifically to breast cancer (Ramirez *et al*, 1999; Westcombe *et al*, 1999). The second covers other common cancers, namely colorectal, gynaecological, lung, upper gastrointestinal and urological (Macdonald *et al*, 2004, 2006; Mitchell *et al*, 2008). Both of these reviews described the extent of patient-mediated and practitioner-mediated delays and identified the factors that may cause such delays. The breast cancer literature was reviewed from 1966 to 1997, and 23 studies met the inclusion criteria; all were assessed as being of sufficient quality for inclusion (Ramirez *et al*, 1999). Literature relating to the other cancers was reviewed from 1970 to 2003, and 54 studies met the inclusion criteria for colorectal cancer (45 sufficient quality), 16 for gynaecological cancers (12 sufficient quality), 8 for lung cancer (4 sufficient quality), 25 for upper gastrointestinal cancers (18 sufficient quality) and 16 for urological cancers (11 sufficient quality). Taken together. therefore, 142 papers were reviewed, of which 113 were deemed of sufficient quality for inclusion in the analyses. Detailed methods of each review are described elsewhere, including the process of determining quality (Westcombe *et al*, 1999; Macdonald *et al*, 2004). This paper reports on and discusses risk factors for patient and primary care practitioner delay. It combines and summarises earlier reported results for breast, colorectal and upper gastrointestinal cancers, with previously unreported results for gynaecological, lung and urological cancers. Only risk factors derived from studies of sufficient quality are presented; these are reported as either increasing or reducing delay or as inconclusive, that is the evidence was neither in support of the factor predominately increasing nor reducing delay, and as such, the direction of influence cannot be determined definitively.

### Terminology

The term ‘delay’ in the cancer literature is used both to refer to time delays and to denote advanced stage at presentation. More recently, authors have tended to avoid use of the word delay, not only because of this confusion, but also because the term implies an active decision for inaction. However, the term is used extensively in the literature and as such is impossible to avoid in a report of this nature. Any reference to delay in this paper refers to time delay.

## Results

### Risk factors for patient delay

#### Demographic factors

Several demographic factors have been studied in relation to their association with presentation for cancer symptoms.

*Age* Older patient age was found to be a risk factor for delayed presentation with symptoms of breast cancer (Ramirez *et al*, 1999), but was unrelated to delay for colorectal cancer (Mitchell *et al*, 2008), urological cancers (Thornhill *et al*, 1987; Samet *et al*, 1988; Mansson *et al*, 1993; Risberg *et al*, 1996) and lung cancer (Worden and Weisman, 1975; Mor *et al*, 1990; Risberg *et al*, 1996). There was no conclusive evidence in relation to the influence of age on presentation with symptoms of upper gastrointestinal (Macdonald *et al*, 2006) or gynaecological cancers (Fruchter *et al*, 1980; Franceschi *et al*, 1983; Fowler *et al*, 1984; Smith and Anderson, 1985; Dhamija *et al*, 1993; Andersen *et al*, 1995; Coates *et al*, 1996; Risberg *et al*, 1996; Jones and Joura, 1999; Goff *et al*, 2000) (Tables 1 and 3, 4 and 5).

*Gender* Overall, men were found to delay longer than women in presenting with bladder cancer (Mommsen *et al*, 1983; Mansson *et al*, 1993; Risberg *et al*, 1996) and with other urological cancers (Risberg *et al*, 1996). There was little evidence of any association between sex and time to presentation for either upper or lower gastrointestinal cancers (Macdonald *et al*, 2006; Mitchell *et al*, 2008) or for lung cancer (Worden and Weisman, 1975; Mor *et al*, 1990; Risberg *et al*, 1996; Bowen and Rayner, 2002).

*Socio-economic status and education* Lower socio-economic status was associated with increased delay by patients presenting with symptoms of upper gastrointestinal cancers (Macdonald *et al*, 2006) and by men with prostate cancer (Hackett *et al*, 1973; Samet *et al*, 1988; Fitzpatrick *et al*, 1998). However, there was no overall relationship between socio-economic status and delay for colorectal cancer (Mitchell *et al*, 2008), gynaecological cancers (Fruchter *et al*, 1980; Fowler *et al*, 1984; Andersen *et al*, 1995; Coates *et al*, 1996; Goff *et al*, 2000) or lung cancer (Hackett *et al*, 1973; Worden and Weisman, 1975; Mor *et al*, 1990). Similarly, although lower educational attainment was associated with greater delay for patients with breast (Ramirez *et al*, 1999) and colorectal cancers (Mitchell *et al*, 2008), it was not related to presentation for any of the urological (Mansson *et al*, 1993; Risberg *et al*, 1996) or gynaecological cancers (Fruchter *et al*, 1980; Franceschi *et al*, 1983; Dhamija *et al*, 1993; Andersen *et al*, 1995; Risberg *et al*, 1996) or for lung cancer (Mor *et al*, 1990; Risberg *et al*, 1996).

*Ethnicity* Non-white ethnic origin was a risk factor for longer delay in presenting with breast (Ramirez *et al*, 1999) and urological cancers (bladder and prostate) (Samet *et al*, 1988; Prout *et al*, 2000), but non-white patients were found to have a shorter time to presentation for stomach cancer (Macdonald *et al*, 2006). No association was found between ethnicity and time to presentation for gynaecological cancers (cervical and uterine) (Fruchter *et al*, 1980; Fowler *et al*, 1984; Coates *et al*, 1996).

*Marital status* Marital status or living with a partner was found to be unrelated to presentation patterns for breast, gynaecological, lung, upper gastrointestinal and urological cancers (Worden and Weisman, 1975; Franceschi *et al*, 1983; Thornhill *et al*, 1987; Mor *et al*, 1990; Mansson *et al*, 1993; Andersen *et al*, 1995; Fitzpatrick *et al*, 1998; Ramirez *et al*, 1999; Macdonald *et al*, 2006). It was not possible to draw a definitive conclusion in relation to its influence on colorectal cancer (Mitchell *et al*, 2008).

#### Clinical factors

*Symptom type* The type of symptoms with which patients present has a marked impact on presenting behaviour (Tables 1 and 3, 4 and 5). Women diagnosed with breast cancer were more likely to delay if they had an atypical symptom, that is one that did not include a breast lump (Ramirez *et al*, 1999). Across the cancer groups, patients were typically less likely to delay if they experienced a more serious symptom, such as pain, or an alarming symptom, such as bleeding. This was found to be the case for upper gastrointestinal cancers (Macdonald *et al*, 2006) and gynaecological cancers (Smith and Anderson, 1985, 1987; Menon *et al*, 1991; Coates *et al*, 1996; Goff *et al*, 2000) for pain associated with colorectal cancer (Mitchell *et al*, 2008) and for bleeding associated with urological cancers (bladder and prostate) (Mansson *et al*, 1993; Fitzpatrick *et al*, 1998; Ho *et al*, 1998). Conversely, pain was a risk factor for increased delay in patients with urological cancers (Hackett *et al*, 1973; Attard, 1985; Mansson *et al*, 1993; Fitzpatrick *et al*, 1998), whereas the evidence for lung cancer was inconclusive (Hackett *et al*, 1973; Worden and Weisman, 1975). Perhaps, unsurprisingly, the onset of vague, non-specific, more common or multiple symptoms was likely to increase delay (Andersen *et al*, 1995; Goff *et al*, 2000; Fitch *et al*, 2002; Mitchell *et al*, 2008). For the majority of cancer groups studied, patients were more likely to present when symptoms were, or became, more incapacitating and impacted on daily life and activities (Hackett *et al*, 1973; Menon *et al*, 1991; Andersen *et al*, 1995; Sanden *et al*, 2000; Macdonald *et al*, 2006; Mitchell *et al*, 2008).

*Co-morbidity* The presence of co-existing morbidity was associated with reduced delay in patients with upper gastrointestinal cancers (Macdonald *et al*, 2006) and colorectal cancer (Mitchell *et al*, 2008). However, this was found to be unrelated to presenting behaviour for lung cancer (Mor *et al*, 1990). There was evidence that regular visits to medical practitioners, including attendance for routine screening, was associated with shorter delay in patients with gynaecological cancers (uterine and cervical) (Menon *et al*, 1991; Coates *et al*, 1996) and prostate cancer (Samet *et al*, 1988; Fitzpatrick *et al*, 1998). The impact of a personal or family history of cancer on help-seeking behaviour was investigated across several sites, and in the main, there was no conclusive evidence as to its impact. However, for patients with prostate cancer, having had a previous cancer diagnosis (Samet *et al*, 1988) or having a relative with cancer (Hackett *et al*, 1973; Fitzpatrick *et al*, 1998) was typically associated with reduced time to presentation. Furthermore, there was evidence of a positive relationship between increased delay and having previously had a normal test result or the condition diagnosed as benign (Fruchter *et al*, 1980; Smith and Anderson, 1985).

#### Psychosocial factors

*Awareness and interpretation of symptoms* Symptom awareness, and more particularly patients’ interpretation of symptoms, was a commonly reported theme. Non-recognition of the seriousness of symptoms, sometimes related to lack of knowledge about the disease, was the predominant risk factor for delay reported across all cancer sites (Worden and Weisman, 1975; Fruchter *et al*, 1980; Bosl *et al*, 1981; Attard, 1985; Smith and Anderson, 1985, 1987; Cochran *et al*, 1986; Mor *et al*, 1990; Menon *et al*, 1991; Mansson *et al*, 1993; Andersen *et al*, 1995; Coates *et al*, 1996; Ajayi and Adewole, 1998; Gascoigne *et al*, 1999; Ramirez *et al*, 1999; de Nooijer *et al*, 2001; Bowen and Rayner, 2002; Fitch *et al*, 2002; Khadra and Oakeshott, 2002; Kidanto *et al*, 2002; Macdonald *et al*, 2006; Mitchell *et al*, 2008) (Tables 1 and 3, 4 and 5). Delay was often related to patients adopting a ‘wait and see’ approach, denying or redefining their symptoms in relation to benign disease or self-diagnosing and self-medicating before presentation to a practitioner (Macdonald *et al*, 2006; Mitchell *et al*, 2008).

*Emotional response* Concern related to recognition of a potential cancer symptom was also important in the decision to present. Fear that a symptom was indicative of cancer, or fear of investigation, of treatment, or of powerlessness were also found to be factors in increasing time to presentation for upper and lower gastrointestinal cancers (Macdonald *et al*, 2006; Mitchell *et al*, 2008), urological cancers (Hackett *et al*, 1973; Fitzpatrick *et al*, 1998; Ho *et al*, 1998; de Nooijer *et al*, 2001; Sanden *et al*, 2000), gynaecological cancers (Fruchter *et al*, 1980; Smith and Anderson, 1985, 1987) and lung cancer (Hackett *et al*, 1973; Worden and Weisman, 1975; Mor *et al*, 1990). Similarly, embarrassment about symptoms resulted in longer delay for patients with colorectal (Mitchell *et al*, 2008) and urological cancers (testicular and prostate) (Fitzpatrick *et al*, 1998; Gascoigne *et al*, 1999).

*Support* Social support and the availability of advice were influential factors in patients’ decisions to present with cancer symptoms. Patients with breast cancer, who did not disclose their symptoms within a week to someone close to them, were more likely to delay help seeking (Ramirez *et al*, 1999). For patients with colorectal cancer, social networks were identified as a potentially important factor in reducing delay, when patients either sought advice from or made decisions based on the experience of others (Mitchell *et al*, 2008). In keeping with this, lower levels of social support were found to be associated with increased delay in women with endometrial cancer (Cochran *et al*, 1986) (Table 1). However, the availability of social support was unrelated to delay for lung, upper gastrointestinal and urological cancers (Samet *et al*, 1988; Mor *et al*, 1990; Bowen and Rayner, 2002; Sanden *et al*, 2000; Macdonald *et al*, 2006).

### Risk factors for practitioner delay

#### Patient demographic factors

There was some evidence relating to the impact of certain patient characteristics on practitioners’ referral behaviour (Tables 2, 3, 4 and 5).

*Age and gender* Older patients were referred more quickly for symptoms of breast, upper gastrointestinal and colorectal cancers (Ramirez *et al*, 1999; Macdonald *et al*, 2006; Mitchell *et al*, 2008). However, younger patients experienced less delay in referral when presenting with symptoms related to one of the urological cancers (Mansson *et al*, 1993; Risberg *et al*, 1996) or to lung cancer (Risberg *et al*, 1996). There was no conclusive evidence on the direction of effect for patient age and practitioner delay for gynaecological cancers (Fruchter *et al*, 1980; Fowler *et al*, 1984; Risberg *et al*, 1996). Men with upper gastrointestinal cancers (Macdonald *et al*, 2006) and urological cancer (bladder) (Mommsen *et al*, 1983; Mansson *et al*, 1993) were less likely than women to have experienced delayed referral, although the evidence relating to colorectal cancer was inconclusive (Mitchell *et al*, 2008).

*Socio-economic status, education and ethnicity* Patients from lower socio-economic groups experienced a shorter wait to referral for upper gastrointestinal cancer (Macdonald *et al*, 2006), but greater delay than more affluent patients in referral for colorectal cancer (Mitchell *et al*, 2008). Although socio-economic status was unrelated to referral for symptoms of gynaecological cancers (Fowler *et al*, 1984; Goff *et al*, 2000), lower educational attainment was associated with delayed referral in this group, as it was for patients with symptoms of lung and urological cancers (Risberg *et al*, 1996). There was little evidence on the influence of ethnicity, although non-white women with breast cancer experienced less practitioner delay (Ramirez *et al*, 1999), whereas this was unrelated to referral for gynaecological cancer (cervical) (Fowler *et al*, 1984).

#### Presenting symptom and medical history

As was the case in patient delay, presenting with a breast symptom other than a lump was associated with greater delay by practitioners (Ramirez *et al*, 1999). Typically, pain as a presenting symptom resulted in shorter time to referral for patients with upper gastrointestinal cancers (Macdonald *et al*, 2006) and gynaecological cancer (ovarian) (Goff *et al*, 2000; Fitch *et al*, 2002). This was also found to be the case for bleeding in upper gastrointestinal cancers (Macdonald *et al*, 2006) and urological cancer (bladder) (Mommsen *et al*, 1983; Mansson *et al*, 1993). Patients with co-existing disease were likely to be referred more quickly for symptoms of colorectal cancer (Mitchell *et al*, 2008), but the evidence relating to upper gastrointestinal cancers was inconclusive (Macdonald *et al*, 2006). However, time to referral for colorectal cancer was increased for patients who frequently consulted their general practitioner (Mitchell *et al*, 2008), and the involvement of multiple care providers resulted in delay for patients with gynaecological cancer (ovarian) (Fowler *et al*, 1984; Goff *et al*, 2000). Frequency of attendance was unrelated to referral for upper gastrointestinal cancers (Macdonald *et al*, 2006).

#### Practitioner response

The most commonly identified themes associated with delayed referral across the cancer sites related to initial diagnosis and activity of the practitioner. Misdiagnosis, occurring either through treating patients symptomatically or by relating symptoms to a health problem other than cancer, resulted in increased time to referral for breast, colorectal, gynaecological, upper gastrointestinal and urological cancers (Bosl *et al*, 1981; Attard, 1985; Gascoigne *et al*, 1999; Jones and Joura, 1999; Ramirez *et al*, 1999; Goff *et al*, 2000; Fitch *et al*, 2002; Macdonald *et al*, 2006; Mitchell *et al*, 2008). Although there was support for the existence of a similar pattern in relation to diagnosis of lung cancer (Table 4), the evidence was of relatively poor quality (Pereira *et al*, 1991; Silva *et al*, 1992; Bowen and Rayner, 2002; Koyi *et al*, 2002). Failure to fully or adequately examine patients, use of inappropriate or inadequate tests and receiving or failing to follow-up inconclusive, negative or false negative test results contributed to the delay (Fruchter *et al*, 1980; Bosl *et al*, 1981; Hernes *et al*, 1996; Goff *et al*, 2000; Macdonald *et al*, 2006; Mitchell *et al*, 2008). Similarly, prescribing treatment for benign conditions, such as acid suppression in patients subsequently diagnosed with upper gastrointestinal cancers (Macdonald *et al*, 2006), or antibiotics in patients with testicular cancer (Bosl *et al*, 1981) also increased time to referral. There is some evidence to suggest that appropriate referral and use of referral guidelines is associated with reduced delay for upper gastrointestinal cancers (Macdonald *et al*, 2006) and colorectal cancer (Mitchell *et al*, 2008).

## Discussion

Reasons for delay in presentation by patients with symptoms of cancer and influences on time to referral by practitioners are complex and multi-factorial according to the combined findings of two systematic reviews of the literature on common cancers. The predominant risk factor for patient delay is a lack of interpretation by patients of the serious nature of their symptoms. Across the common cancers, symptom type is predictive of delay in presentation. If a symptom is atypical, or vague in nature, the risk of delayed presentation can be increased. Conversely, if the symptom is more serious or alarming and includes a lump, bleeding or pain, then the risk of delayed presentation is typically reduced. Patients may fail to recognise or appreciate atypical or vague symptoms, which may mediate delayed presentation. Where symptoms are understood and thought to be serious, there is a reduced time to presentation, and whatever the cancer site, the catalyst for presentation is often when a symptom becomes incapacitating or impacts on normal activities, rather than the presence of the symptom alone. However, the difficulty is that common cancer symptoms are often attributable to benign disease. The complexity of this process can be illustrated by considering breast cancer, where there is robust evidence that patients delay less with the well-known symptom of lump, compared with the less recognised non-lump symptoms, which result in greater delay. This contrasts with the evidence for urological cancers, where pain increases delay, perhaps as a result of symptoms being misinterpreted as being due to a benign cause such as cystitis. In other cancers too, such as those of the gastrointestinal tract, potential cancer symptoms can frequently have a benign interpretation.

Patient delays are also influenced by a range of demographic and psychosocial factors. Taken together, lower socio-economic status and education level are risk factors for delayed presentation of several common cancers, but not for others. Similarly, non-white ethnic origin impacts on delayed presentation for some, but not all common cancers. Neither age nor gender is associated with delayed presentation with the exception of breast cancer (older age) and bladder cancer (male sex). Across the common cancers, then demographic risk factors for patient delay seem inconsistent. This may be a consequence of the fact that the cancers have not been equally researched in terms of risk factors for delay. Although the review of the literature for breast cancer was based on 23 studies deemed to be of adequate quality, and the upper and lower gastrointestinal results were based on 18 and 45 studies, respectively, only 11 urological, 12 gynaecological and 4 lung cancer studies were of sufficient quality to be included in the synthesis of evidence. Further, it is possible that some of the factors we have described are not independent of each other; the ways in which they may relate to each other are not clear.

General population surveys in the United Kingdom indicate a widespread lack of awareness of the symptoms of cancer (Grunfeld *et al*, 2002; McCaffery *et al*, 2003; Linsell *et al*, 2008; Robb *et al*, 2009). These low levels of symptom awareness may partly explain why the type of symptom and recognition of the seriousness of symptoms are consistent risk factors for delayed patient presentation. These surveys also suggest that cancer symptom awareness is poorer among those who are less well educated, those with lower socio-economic status and those from black and minority ethnic groups (Wardle *et al*, 2001; Grunfeld *et al*, 2002; Robb *et al*, 2009). Not only are levels of awareness of cancer symptoms low among the general population, but so too is knowledge of the risk factors for developing cancer. Equally, these surveys report that people hold negative beliefs and attitudes about the benefits of seeking medical help for cancer, which include fear, embarrassment, reluctance to bother the general practitioner and nihilism about cancer treatments (Grunfeld *et al*, 2002; Robb *et al*, 2009). These barriers may explain the finding from our review that fear and embarrassment are risk factors for patient delay.

Qualitative research with people diagnosed with cancer further enhances the findings of such surveys. Reported barriers to early help seeking are vague and mild symptoms, absence of pain or lump, belief that the symptom will go away, intermittent symptoms, lack of awareness of cancer risk and previous benign diagnosis (Smith *et al*, 2005). Competing demands and priorities, fears about cancer treatments and anxieties about ‘bothering the doctor’ have also been identified as issues related to delayed presentation in breast cancer (Burgess *et al*, 2001). Qualitative interviews have enabled the identification of eventual triggers to help seeking, such as worsening symptoms, new additional symptoms, the presence of a symptom that is recognised as serious or is affecting daily life and the influence of family and friends (Burgess *et al*, 2001; Smith *et al*, 2005), findings supported by the evidence presented in this paper.

Overall, research into the risk factors for patient delay indicates that presentation with cancer is not a straightforward or linear process. Knowledge of symptoms and risk may be necessary, but not sufficient to determine help seeking for cancer. People's attitudes, beliefs and social context clearly influence the process of medical help seeking. To develop strategies to reduce patient delays, the risk factors for delayed presentation need to be placed into an explanatory framework. This aids understanding of the complexities of the delay process and highlights where and how interventions could be targeted. A framework for understanding patient delay in breast cancer is described by Bish *et al* (2005). This model incorporates symptom appraisal, attitudes towards help-seeking and translating intentions to seek help into behaviour. It draws on health psychology models, including self-regulation theory (Leventhal *et al*, 1984), theory of planned behaviour (Ajzen, 1991) and implementation intentions (Gollwitzer, 1999). A similar model (Andersen *et al*, 1995) has been suggested for other cancers. Basing an intervention to promote behaviour change in an explanatory framework informed by psychological theory and empirical evidence increases the likelihood that it will be effective (Bish *et al*, 2005; Serlachius and Sutton, 2009).

To impact on presentation with cancer symptoms, we need a greater understanding of the psychological and sociological factors influencing patients’ help-seeking behaviour. In addition, we need to devise culturally sensitive strategies, not only to improve awareness of cancer, but also to aid interpretation of symptom seriousness by patients. Ideally, an intervention to reduce delayed presentation of cancer would promote early help-seeking behaviour by people with a high cancer risk, but would not promote anxiety among those at low risk. It is important that patients are neither made unnecessarily anxious, nor should general practitioners be overburdened by consultations with ‘the worried well’. The demographic risk factors for patient delay and low cancer awareness suggest the population groups that we should target with interventions to promote early presentation. The evidence suggests a general focus on populations that are socially deprived and less educated, as well as those from black and minority ethnic groups. Interventions related to breast cancer should be targeted at older women. However, further research is needed to clarify relevant risk factors for delayed time to presentation for some particularly under-researched cancers, such as lung and prostate.

When people do present to their doctor with potential cancer symptoms, timely investigation and onwards referral are clearly important. The reviews reported here suggest that patients with particular demographic profiles are likely to experience practitioner delay, including, for some cancer sites, women and those with lower educational attainment. There is also evidence that vague and atypical symptoms are not only associated with patient delays, but also with practitioner delays. The challenge is that among the hundreds of patients with potential cancer symptoms that every general practitioner sees each year, only eight of these will actually have the disease. This review also suggests the need for effective use of referral guidelines for general practitioners, as well as better use of, and access to, diagnostic services.

## Conflict of interest

The authors declare no conflict of interest.

## Figures and Tables

**Table 1:** Risk factors for patient-mediated delay

**Risk factor**	**Breast**	**Upper GI^a^**	**Colorectal**	**Uro^b^**	**Gyn^c^**	**Lung**
*Demographic*						
Age (older)	↑	⊙	=	=	⊙	=
Sex (male)	N/A	=	=	↑	N/A	=
Socio-economic status (lower)	⊙	↑	=	↑	=	=
Education level (lower)	↑	⊙	↑	=	=	=
Ethnicity (non-white origin)	↑	↓	⊙	↑	=	○
Marital status (married/co-habiting)	=	=	⊙	=	=	=
						
*Clinical*						
Symptom type	↑	↑	↑	↑	↑	⊙
Symptom – pain	◊	↓	↓	↑	↓	⊙
Symptom – bleeding	◊	↓	⊙	↓	↓	○
Symptom impacts on daily life	○	↓	↓	↓	↓	↓
Co-morbidity	○	↓	↓	○	○	=
Infrequent care seeking	○	○	⊙	↑	↑	○
Personal/family history of cancer	○	⊙	⊙	↓	○	⊙
						
*Psychosocial*						
Non-recognition of symptom seriousness	↑	↑	↑	↑	↑	↑
Fear	○	↑	↑	↑	↑	↑
Embarrassment	○	○	↑	↑	○	○
Social support/advice	↓	=	↓	=	↓	=

Abbreviation: GI=gastrointestinal.

Key: ↑ increases delay; ↓ reduces delay; = no impact on delay; ⊙ inconclusive evidence; ○ lacking evidence; ◊ any non-lump symptom.

Risk factor included only if supported by studies providing *strong* or *moderate* levels of evidence (*Sources*: Ramirez *et al*, 1999; Macdonald *et al*, 2004, 2006; Mitchell *et al*, 2008).

a Duodenum, oesophagus, pancreas, small intestine, stomach.

b Bladder, kidney, prostate, testes.

c Cervix, endometrium, ovary, uterus, vagina, vulva.

**Table 2:** Risk factors for practitioner-mediated delay

**Risk factor**	**Breast**	**Upper GI^a^**	**Colorectal**	**Uro^b^**	**Gyn^c^**	**Lung**
*Patient demographics*						
Age (older)	↓	↓	↓	↑	⊙	↑
Sex (male)	N/A	↓	⊙	↓	N/A	○
Socio-economic status (lower)	○	↓	↑	○	=	○
Education level (lower)	○	○	○	↑	↑	↑
Ethnicity (non-white origin)	↓	○	○	○	=	○
						
*Presenting symptom and history*						
Symptom – pain	◊	↓	⊙	○	↓	○
Symptom – bleeding	◊	↓	⊙	↓	○	○
Co-morbidity	○	⊙	↓	○	○	○
Frequent care seeking/multiple providers	○	=	↑	○	↑	○
						
*Practitioner response*						
Initial misdiagnosis	↑	↑	↑	↑	↑	○
Inadequate examination/inappropriate tests	○	↑	↑	↑	↑	○
Treatment for benign condition	○	↑	○	↑	○	○
Use of referral guidelines	○	↓	↓	○	○	○

Abbreviation: GI=gastrointestinal.

Key: ↑ increases delay; ↓ reduces delay; = no impact on delay; ⊙ inconclusive evidence; ○ lacking evidence; ◊ any non-lump symptom.

Risk factor included only if supported by studies providing *strong* or *moderate* levels of evidence (*Sources*: Ramirez *et al*, 1999; Macdonald *et al*, 2004, 2006; Mitchell *et al*, 2008).

a Duodenum, oesophagus, pancreas, small intestine, stomach.

b Bladder, kidney, prostate, testes.

c Cervix, endometrium, ovary, uterus, vagina, vulva.

**Table 3:** Risk factors associated with delay for gynaecological cancers

**Author(s)**	**Location**	**Study type**	**Participants**	**Cancer site**	**Factors which increase delay**	**Factors which reduce delay**	**No impact on delay**	**Evidence**
*Patient-associated factors*								
Fruchter *et al* (1980)	NY, USA	Prospective observational	97 patients	Cervix	Non-recognition of symptom seriousness; previous normal test result; fear		Age; ethnicity; education; income; place of birth	Moderate
Franceschi *et al* (1983)	Italy	Prospective observational	173 patients (aged 33–84; median 59)	Endometrium	Age – younger; pre-menopausal women	Increased patient awareness^a^	Marital status; education; parity; BMI; contraception	Strong
Fowler *et al* (1984)	NC, USA	Retrospective observational	271 patients	Cervix			Age; ethnicity; insurance cover	Moderate
Smith and Anderson (1985)	IA, USA	Prospective observational	82 patients (aged 20–54)	Ovary	Non-recognition of symptom seriousness; re-appearance of previous benign condition; fear	Symptom type – abdominal pain or swelling, irregular bleeding; age – older		Strong
Cochran *et al* (1986)	CA, USA	Prospective observational	37 patients (48–72; median 64)	Endometrium	Attributing symptoms to menopause; marital dissatisfaction; lower social support	Recognition of symptom seriousness (attributing to cancer); longer time since menopause		Moderate
Smith and Anderson (1987)	IA, USA	Prospective observational	98 patients (aged 20–54; median 49)	Endometrium	Non-recognition of symptom seriousness; fear	Symptom type – irregular bleeding, discharge, abdominal pain		Strong
Menon *et al* (1991)	India	Observational	117 patients	Cervix	Non-recognition of symptom seriousness; symptom type – irregular bleeding, vaginal discharge; lack of attendance for check-ups	Symptom type – pus discharge, continuous bleeding		Moderate
Dhamija *et al* (1993)	India	Cross-sectional	1411 ever-married women	Cervix	Lack of patient awareness^a^	Age – younger; education level – higher^a^		Insufficient
Andersen *et al* (1995)	OH, USA	Prospective observational	34 patients (aged 24–75; mean 50)	Endometrium, vulva, ovary, vagina	Age – older; innocuous or diffuse symptoms; attributing symptoms to normal life circumstances	Symptom becomes more prominent/serious	Marital status; education; employment; family circumstances; religion	Strong
Coates *et al* (1996)	GA, LA, CA, USA	Prospective observational	331 patients (aged 20–79)	Uterus	Infrequent care seeking; income – lower; marital status – divorced; occupation – blue collar/service; no health insurance; smoker	Recognition of symptom seriousness; age – older; symptom type – vaginal bleeding	Ethnicity	Moderate
Risberg *et al* (1996)	Norway	Cross-sectional	252 cancer patients (mean age 58; 52% men, 48% women), 5% with gynaecological cancer	All			Age; education level	Strong
Ajayi and Adewole (1998)	Nigeria	Cross-sectional	254 women (aged 20–65)	Cervix	Lack of patient awareness^a^			Insufficient
Jones and Joura (1999)	New Zealand	Retrospective observational	102 patients (aged 36–94)	Vulva	Lack of patient awareness^a^		Age	Insufficient
Goff *et al* (2000)	USA; Canada	Observational	1725 patients (aged 18–84; median 52)	Ovary	Ignoring symptoms; multiple symptoms	Age – older	Health insurance; symptom type	Insufficient
Fitch *et al* (2002)	Toronto, Canada	Qualitative interviews	18 women (aged 35–73; mean 53)	Ovary	Non-recognition of symptom seriousness; symptom type – vague			Strong
Kidanto *et al* (2002)	Tanzania	Case–control	267 patients, 89 with cancer (mean age 49); 178 controls (mean age 46)	Cervix	Non-recognition of symptom seriousness; lack of knowledge			Moderate
								
*Practitioner-associated factors*								
Fruchter *et al* (1980)	NY, USA	Prospective observational	97 women	Cervix	Inconclusive test results; inadequate examination; failure to follow-up negative result; patient age – younger			Moderate
Fowler *et al* (1984)	NC, USA	Retrospective observational	271 patients	Cervix	Involvement of several physicians		Age; ethnicity; insurance cover	Moderate
Risberg *et al* (1996)	Norway	Cross-sectional	252 cancer patients (mean age 58; 52% men, 48% women), 5% with gynaecological cancer	All		Patient age – younger; patient education level – higher		Strong
Jones and Joura (1999)	New Zealand	Retrospective observational	102 patients (aged 36–94)	Vulva	Initial misdiagnosis^a^			Insufficient
Goff *et al* (2000)	USA; Canada	Observational	1725 patients (aged 18–84; median 52)	Ovary	Multiple providers of care; incomplete/inappropriate examination; initial misdiagnosis; multiple symptoms		Type of physician initially seen; health insurance; symptom type	Insufficient
Fitch *et al* (2002)	Canada	Qualitative interviews	18 women (aged 35–73; mean 53)	Ovary	Symptom type – vague; misinterpretation of symptoms	Symptom type – pain		Strong

Abbreviation: BMI=body mass index.

a Study infers findings.

**Table 4:** Risk factors associated with delay for lung cancer

**Author(s)**	**Location**	**Study type**	**Participants**	**Cancer site**	**Factors which increase delay**	**Factors which reduce delay**	**No impact on delay**	**Evidence**
*Patient-associated factors*								
Hackett *et al* (1973)	MA, USA	Prospective observational	563 patients (aged 17–91; mean 62; 46% men, 54% women), 6% with lung cancer	Lung	Symptom type – pain; social class – lower; procrastination; worry over health; family history	Worry; incapacitated by symptoms; acknowledgement of cancer		Strong
Worden and Weisman (1975)	MA, USA	Prospective observational	125 patients (aged 19–59, 38% men, 62% women), 18% with lung cancer	Lung	Non-recognition of symptom seriousness; denial; powerlessness; cancer site – lung compared with breast, Hodgkin	Cancer site – lung compared with melanoma	Age; sex; marital status; socio-economic status; family history; presenting symptoms	Strong
Mor *et al* (1990)	RI, USA	Prospective observational	625 patients (aged 45–90; 31% men, 69% women), 19% with lung cancer	Lung	Non-recognition of symptom seriousness; symptom type – nagging cough; belief that symptoms would ‘go away’; fear of cancer		Age; sex; marital status; education; socio-economic status; social support; co-morbidity	Strong
Risberg *et al* (1996)	Norway	Cross-sectional	252 cancer patients (mean age 58; 52% men, 48% women), 16% with lung cancer	Lung		Cancer site – lung compared with GI, urological, Hodgkin; sex – female	Age; education	Strong
Bowen and Rayner (2002)	England	Prospective observational	37 patients, 51% men (aged 45–80; mean 65), 49% women (aged 44–90; mean 67)	Lung	Non-recognition of symptom seriousness; lack of awareness of symptoms	Sex – female; advice from social network		Insufficient
Koyi *et al* (2002)	Sweden	Prospective observational	134 patients, 63% men (aged 48–90, mean 72); 37% women (aged 35–90, mean 70)	Lung	Lack of awareness; difficulty in accessing primary care^a^			Insufficient
								
*Practitioner-associated factors*								
(Pereira *et al* (1991))	Brazil	Prospective observational	100 patients	Lung	Lack of awareness; inadequate tests^a^			Insufficient
(Silva *et al* (1992))	Brazil	Prospective observational	300 patients	Lung		Improved GP awareness^a^		Insufficient
Risberg *et al* (1996)	Norway	Cross-sectional	252 cancer patients (mean age 58: 52% men, 48% women), 16% with lung cancer	Lung		Patient age – younger; patient education level – higher		Strong
Bowen and Rayner (2002)	England	Prospective observational	37 patients, 19 men (mean age 65), 18 women (mean age 67)	Lung	Lack of symptom awareness			Insufficient
Koyi *et al* (2002)	Sweden	Prospective observational	134 patients, 63% men (aged 48–90, mean 72); 37% women (aged 35–90, mean 70)	Lung	Lack of suspicion^a^	Low threshold for referral^a^		Insufficient

a Study infers findings; (*non-English language paper*).

**Table 5:** Risk factors associated with delay for urological cancers

**Author(s)**	**Location**	**Study type**	**Participants**	**Cancer site**	**Factors which increase delay**	**Factors which reduce delay**	**No impact on delay**	**Evidence**
*Patient-associated factors*								
Hackett *et al* (1973)	MA, USA	Prospective observational	563 patients (aged 17–91; mean 62, 46% men, 54% women), 9% with prostate cancer	Prostate	Symptom type – pain; social class – lower; procrastination; worry over health; family history	Worry; incapacitated by symptoms; acknowledgement of cancer		Strong
Bosl *et al* (1981)	MN, USA	Retrospective observational	335 patients (mean age 31)	Testicular	Non-recognition of symptom seriousness			Moderate
Mommsen *et al* (1983)	Denmark	Prospective observational	212 patients (mean age 66; 78% men, 22% women)	Bladder	Symptom type – cystitis	Sex – female		Moderate
Attard (1985)	England	Retrospective observational	23 patients	Testicular	Non-recognition of symptom seriousness; symptom type – painless			Insufficient
Thornhill *et al* (1987)	Ireland	Retrospective observational	217 patients	Testicular	Presence of atypical symptoms; symptom type – dragging sensation	Improved patient awareness^a^	Age; marital status; area of residence	Strong
Samet *et al* (1988)	NM, USA	Prospective observational	800 patients, (aged 65–100, mean 72), 32% with prostate cancer	Prostate	Site – prostate compared with breast and colorectal; ethnicity – white Hispanic	Previous cancer diagnosis; regular check-ups	Age; income; availability of vehicle; social support; participation in screening	Strong
Mansson *et al* (1993)	Sweden	Cross-sectional	343 patients (aged 27–94; 77% men, 23% women)	Bladder	Non-recognition of symptom seriousness symptom type – urgency, pain	Symptom type – haematuria	Age; sex; education; marital status	Strong
Hernes *et al* (1996)	Norway	Retrospective observational	352 patients (aged 15–83, median 32)	Testicular	Lack of patient awareness^a^	Cancer type – non-seminoma		Insufficient
Risberg *et al* (1996)	Norway	Cross-sectional	252 cancer patients (mean age 58; 52% men, 48% women), 15% with urological cancer	Testicular, prostate, bladder, kidney	Cancer site – kidney, bladder, prostate	Cancer site – testicular; sex – female	Age; education level	Strong
Fitzpatrick *et al* (1998)	Ireland	Cross-sectional	280 randomly selected men, (aged 40–69, mean 53)	Prostate	Lack of patient awareness; social class – lower; embarrassment; fear of treatment	Living with female partner; history of urinary tract disease; symptom type – pain, bleeding; saw GP >1 in past year; having a relative with cancer		Moderate
Ho *et al* (1998)	Northern Ireland	Prospective observational	100 haematuria clinic patients (aged 18–97, mean 57; 64% men, 36% women)	Bladder	Fear of cancer; previous haematuria	Improved patient awareness^a^		Insufficient
Gascoigne *et al* (1999)	Wales	Qualitative interviews	11 patients and carers	Testicular	Non-recognition of symptom seriousness; embarrassment			Strong
Prout *et al* (2000)	USA	Prospective observational	497 patients (aged 20–79)	Bladder	Ethnicity – black			Strong
Sanden *et al* (2000)	Sweden	Qualitative interviews	21 patients (aged 20–49)	Testicular	Symptom type – intermittent symptoms; adopting a ‘wait and see’ approach’; fear of cancer diagnosis	Symptom impacting on everyday activity; advice from social network		Insufficient
de Nooijer *et al* (2001)	The Netherlands	Qualitative interviews	23 patients (mean age 52; 43% men, 57% women), 22% with testicular cancer, 10 GPs	Testicular	Non-recognition of symptom seriousness; fear of cancer	Fear of cancer; trust in GP		Strong
Khadra and Oakeshott (2002)	England	Cross-sectional	202 men; (aged 18–50, mean 32)	Testicular	Lack of patient awareness^a^			Insufficient
								
*Practitioner-associated factors*								
Bosl *et al* (1981)	MN, USA	Retrospective observational	335 patients (mean age 31)	Testicular	Lack of awareness; initial misdiagnosis; inadequate examination; inappropriate treatment			Moderate
Mommsen *et al* (1983)	Denmark	Prospective observational	212 patients (mean age 66; 78% men, 22% women)	Bladder	Symptom type – cystitis (especially men); patient sex – female	Symptom type – haematuria (especially men)		Moderate
Attard (1985)	England	Retrospective observational	23 patients	Testicular	Initial misdiagnosis			Insufficient
Mansson *et al* (1993)	Sweden	Cross-sectional	343 patients (aged 27–94; 77% men, 23% women)	Bladder	Symptom type – urgency	Symptom type – haematuria plus pain; patient sex – male; patient age – younger		Strong
Hernes *et al* (1996)	Norway	Retrospective observational	352 patients (aged 15–83, median 32)	Testicular	Lack of awareness; inadequate use of tests^a^			Insufficient
Risberg *et al* (1996)	Norway	Cross-sectional	252 cancer patients (mean age 58; 52% men, 48% women), 15% with urological cancer	Testicular, prostate, bladder, kidney		Patient age – younger; patient education level – higher		Strong
Ho *et al* (1998)	Northern Ireland	Prospective observational	100 haematuria clinic patients (aged 18–97, mean 57; 64% men, 36% women)	Bladder	Inappropriate referral^a^			Insufficient
Gascoigne *et al* (1999)	Wales	Qualitative interviews	11 patients and carers	Testicular	Initial misdiagnosis			Strong

Abbreviation: GP=general practitioner.

a Study infers findings.

## References

[bib1] Ajayi I, Adewole I (1998) Knowledge and attitudes of general outpatient attendants in Nigeria to cervical cancer. Central Afr J Med 44(2): 41–43 9675971

[bib2] Ajzen I (1991) The theory of planned behavior. Organ Behav Hum Decis Process 50(2): 179–211

[bib3] Andersen BL, Cacioppo JT, Roberts DC (1995) Delay in seeking a cancer diagnosis: delay stages and psychophysiological comparison processes. Br J Soc Psych 34: 33–52 10.1111/j.2044-8309.1995.tb01047.x 7735731

[bib4] Attard GJ (1985) Delay in treatment of testicular tumours in the army. J R Army Med Corps 131: 140–141 4087240 10.1136/jramc-131-03-04

[bib5] Bish A, Ramirez A, Burgess C, Hunter M (2005) Understanding why women delay in seeking help for breast cancer symptoms. J Psychosoma Res 58(4): 321–326 10.1016/j.jpsychores.2004.10.007 15992567

[bib6] Bosl GJ, Goldman A, Lange PH, Vogelzang NJ, Fraley EE, Levitt SH, Kennedy BH (1981) Impact of delay in diagnosis on clinical stage of testicular cancer. Lancet 2: 970–972 6117736 10.1016/s0140-6736(81)91165-x

[bib7] Bowen EF, Rayner CFJ (2002) Patient and GP led delays in the recognition of symptoms suggestive of lung cancer. Lung Cancer 37: 227–228 12140147 10.1016/s0169-5002(02)00143-5

[bib8] Burgess CC, Hunter MS, Ramirez AJ (2001) A qualitative study of delay among women reporting symptoms of breast cancer. Br J Gen Pract 51: 967–971 11766868 PMC1314188

[bib9] Cancer Research UK (2009) Cancer cure rates improving in Europe. http://info.cancerresearchuk.org/news/archive/newsarchive/2009/march/19090650. Accessed 27 August 2009

[bib10] Coates RJ, Click LA, Harlan LC, Robboy S, Barrett RJ, Eley JW, Reynolds P, Chen VW, Darity WA, Blacklow RS, Edwards BK (1996) Differences between Black and White patients with cancer of the uterine corpus in interval from symptom recognition to initial medical consultation (United States). Cancer Causes Control 7: 328–336 8734826 10.1007/BF00052938

[bib11] Cochran SD, Hackett NF, Berek J (1986) Correlates of delay in seeking treatment for endometrial cancer. J Psychosom Obstet Gynaecol 5: 245–252

[bib12] de Nooijer J, Lechner L, de Vries H (2001) Help-seeking behaviour for cancer symptoms: perceptions of patients and general practitioners. Psychooncology 10(6): 469–478 11747059 10.1002/pon.535

[bib13] Dhamija S, Sehgal A, Luthra UK, Sehgal K (1993) Factors associated with awareness and knowledge of cervical cancer in a community: implication for health education programmes in developing countries. J R Soc Health 113(4): 184–186 8410910 10.1177/146642409311300407

[bib14] Fitch M, Deane K, Howell D, Gray RE (2002) Women's experiences with ovarian cancer: reflections on being diagnosed. Can Oncol Nurs J 12(3): 152–168 12271917 10.5737/1181912x123152159

[bib15] Fitzpatrick P, Corcoran N, Fitzpatrick JM (1998) Prostate cancer: how aware is the public? Br J Urol 82: 43–48 9698661 10.1046/j.1464-410x.1998.00685.x

[bib16] Fowler WC, Freeman AC, Hulka BS, Kaluzny AD, O’Keefe SP, Symons MJ, Lee YY (1984) Delays in cervical cancer treatment: an assessment of patient and provider characteristics. Prog Clin Biol Res 156: 265–274 6473435

[bib17] Franceschi S, La Vecchia C, Gallus G, DeCarli A, Colombo E, Mangioni C, Tognoni G (1983) Delayed diagnosis of endometrial cancer in Italy. Cancer 51(6): 1176–1178 6821871 10.1002/1097-0142(19830315)51:6<1176::aid-cncr2820510634>3.0.co;2-o

[bib18] Fruchter RG, Boyce J, Hunt M, Sillman F, Medhat I (1980) Invasive cancer of cervix: failures in prevention II. Delays in diagnosis. NY State J Med 80(6): 913–917 6931294

[bib19] Gascoigne P, Mason MD, Roberts E (1999) Factors affecting presentation and delay in patients with testicular cancer: results of a qualitative study. Psychooncology 8: 144–154 10335558 10.1002/(SICI)1099-1611(199903/04)8:2<144::AID-PON349>3.0.CO;2-P

[bib20] Goff BA, Mandel L, Muntz HG, Melancon CH (2000) Ovarian carcinoma diagnosis. Results of a national ovarian cancer survey. Cancer 89(10): 2068–2075 11066047 10.1002/1097-0142(20001115)89:10<2068::aid-cncr6>3.0.co;2-z

[bib21] Gollwitzer PM (1999) Implementation intentions: strong effects of simple plans. Am Psychol 54: 493–503

[bib22] Grunfeld EA, Ramirez AJ, Hunter MS, Richards MA (2002) Women's knowledge and beliefs regarding breast cancer. Br J Cancer 86: 1373–1378 11986766 10.1038/sj.bjc.6600260 PMC2375381

[bib23] Hackett TP, Cassem NH, Raker JW (1973) Patient delay in cancer. N Engl J Med 289(1): 14–20 4708950 10.1056/NEJM197307052890104

[bib24] Hernes EH, Harstad K, Fossa SD (1996) Changing incidence and delay of testicular cancer in southern Norway. Eur Urol 30: 349–357 8931969 10.1159/000474195

[bib25] Ho ETS, Johnston SR, Keane PF (1998) The haematuria clinic – referral patterns in Northern Ireland. Ulster Med J 67(1): 25–28 PMC2448693 9652195

[bib26] Jones RW, Joura EA (1999) Analyzing prior clinical events at presentation in 102 women with vulvar carcinoma. Evidence of diagnostic delays. J Reprod Med 44: 766–768 10509298

[bib27] Khadra A, Oakeshott P (2002) Pilot study of testicular cancer awareness and testicular self-examination in men attending two South London general practices. Fam Pract 19(3): 294–296 11978722 10.1093/fampra/19.3.294

[bib28] Kidanto HL, Kilewo CD, Moshiro C (2002) Cancer of the cervix: knowledge and attitudes of female patients admitted at Muhimbili National Hospital, Der Es Salaam. East Afr Med J 79(9): 467–475 12625687 10.4314/eamj.v79i9.9118

[bib29] Koyi H, Hillerdal G, Branden E (2002) Patient's and doctors’ delays in the diagnosis of chest tumors. Lung Cancer 35: 53–57 11750713 10.1016/s0169-5002(01)00293-8

[bib30] Leventhal H, Nerenz D, Steele DJ (1984) Illness representations and coping with health threats. In Handbook of Psychology and Health Baum A, Taylor SE Singer JE (eds), IV. Lawrence Erlbaum: Hillsdale, NJ

[bib31] Linsell L, Burgess CC, Ramirez AJ (2008) Breast cancer awareness among older women. Br J Cancer 99: 1221–1225 18813307 10.1038/sj.bjc.6604668 PMC2570528

[bib32] Macdonald S, Macleod U, Campbell NC, Weller D, Mitchell E (2006) Systematic review of factors influencing patient and practitioner delay in diagnosis of upper gastrointestinal cancer. Br J Cancer 94: 1272–1280 16622459 10.1038/sj.bjc.6603089 PMC2361411

[bib33] Macdonald S, Macleod U, Mitchell E, Weller D, Campbell N, Mant D (2004) Factors Influencing Patient and Primary Care Delay in the Diagnosis of Cancer: A Database of Existing Research and Its Implications for Future Practice. Final report to the UK Department of Health. University of Glasgow: Glasgow

[bib34] Mansson A, Anderson H, Colleen S (1993) Time lag to diagnosis of bladder cancer – influence of psychosocial parameters and level of health-care provision. Scand J Urol Nephrol 27: 363–369 8290917 10.3109/00365599309180448

[bib35] McCaffery K, Wardle J, Waller J (2003) Knowledge, attitudes, and behavioural intentions in relation to the early detection of colorectal cancer in the United Kingdom. Prevent Med 36(5): 525–535 10.1016/s0091-7435(03)00016-1 12689797

[bib36] Mitchell E, Macdonald S, Campbell NC, Weller D, Macleod U (2008) Influences on pre-hospital delay in the diagnosis of colorectal cancer: a systematic review. Br J Cancer 98: 60–70 18059401 10.1038/sj.bjc.6604096 PMC2359711

[bib37] Menon R, Sehgal A, Singh V, Murthy NS, Luthra UK (1991) Medical attention-seeking behaviour of cervical cancer patients. Implications for cervical cancer control in developing countries. Cancer J 4(3): 202–205

[bib38] Mommsen S, Aagaard J, Sell A (1983) Presenting symptoms, treatment delay and survival in bladder cancer. Scand J Urol Nephrol 17(2): 163–167 6612234 10.3109/00365598309180162

[bib39] Mor V, Masterson-Allen S, Goldberg R, Guadagnoli E, Wool MS (1990) Pre-diagnostic symptom recognition and help seeking among cancer patients. J Community Health 15(4): 253–266 2212095 10.1007/BF01350291

[bib40] Nichols S, Waters WE, Fraser JD, Wheeller MJ, Ingham SK (1981) Delay in the presentation of breast symptoms for consultant investigation. Commun Med 3: 217–225 10.1007/BF02549119 7273693

[bib41] Pereira JR, Ikari FK, Minamoto H, Cassioloi JC (1991) Delay factors in the diagnosis of lung cancer: a public health problem. Revista Paulista de Medicina 109(3): 109–112 1947604

[bib42] Prout GR, Wesley MN, Greenberg RS, Chen VW, Brown CC, Miller AW, Weinstein RS, Robboy SJ, Haynes MA, Blacklow RS, Edwards BK (2000) Bladder cancer. Race differences in extent of disease at diagnosis. Cancer 89(6): 1349–1358 11002231 10.1002/1097-0142(20000915)89:6<1349::aid-cncr20>3.0.co;2-d

[bib43] Ramirez AJ, Westcombe AM, Burgess CC, Sutton S, Littlejohns P, Richards MA (1999) Factors predicting delayed presentation of symptomatic breast cancer: a systematic review. Lancet 353: 1127–1131 10209975 10.1016/s0140-6736(99)02142-x

[bib44] Richards MA, Smith P, Ramirez AJ, Fentiman IS, Rubens RD (1999) The influence on survival of delay in the presentation and treatment of symptomatic breast cancer. Br J Cancer 79: 858–864 10070881 10.1038/sj.bjc.6690137 PMC2362673

[bib45] Risberg T, Sorbye SW, Norum J, Wist EA (1996) Diagnostic delay causes more psychological distress in female than in male cancer patients. Anticancer Res 16(2): 995–999 8687166

[bib46] Robb K, Stubbings S, Ramirez A, Macleod U, Austoker J, Waller J, Hiom S, Wardle J (2009) Public awareness of cancer in Britain: a population-based survey of adults. Br J Cancer 101(Suppl 2): S18–S23 10.1038/sj.bjc.6605386 PMC2790705 19956158

[bib47] Samet JM, Hunt WC, Lerchen ML, Goodwin JS (1988) Delay in seeking care for cancer symptoms: a population-based study of elderly New Mexicans. J Natl Cancer Inst 80(6): 432–438 3367383 10.1093/jnci/80.6.432

[bib48] Sanden I, Larsson US, Eriksson C (2000) An interview study of men discovering testicular cancer. Cancer Nurs 23(4): 304–309 10939178 10.1097/00002820-200008000-00008

[bib49] Serlachius A, Sutton S (2009) Self management and behaviour change: theoretical models. In Chronic Physical Illness: Self Management and Behavioural Interventions, Newman S, Steed L, Mulligan K (eds), pp 48–63. Open University Press: Buckingham

[bib50] Silva PP, Pereira JR, Ikari FK, Minamoto H (1992) Lung cancer and the delay in the diagnosis: analysis of 300 cases. Revista Da Associacao Medica Brasileira 38(3): 145–149 1340364

[bib51] Smith EM, Anderson B (1985) The effects of symptoms and delay in seeking diagnosis on stage of disease at diagnosis among women with cancers of the ovary. Cancer 56(11): 2727–2732 4052947 10.1002/1097-0142(19851201)56:11<2727::aid-cncr2820561138>3.0.co;2-8

[bib52] Smith EM, Anderson B (1987) Symptomatology, delay and stage of disease in endometrial cancer. Cancer Detect Prev 10(3-4): 247–254 3568023

[bib53] Smith LK, Pope C, Botha JL (2005) Patients’ help-seeking experiences and delay in cancer presentation: a qualitative synthesis. Lancet 366: 825–831 16139657 10.1016/S0140-6736(05)67030-4

[bib54] Thornhill JA, Fennelly JJ, Kelly DG, Walsh A, Fitzpatrick JM (1987) Patients’ delay in the presentation of testis cancer in Ireland. Br J Urol 59(5): 447–451 3594102 10.1111/j.1464-410x.1987.tb04844.x

[bib55] Wardle J, Waller J, Brunswick N, Jarvis MJ (2001) Awareness of risk factors for cancer among British adults. Public Health 115(3): 173–174 11429711 10.1038/sj/ph/1900752

[bib56] Westcombe AM, Richards MA, Ramirez AJ, Love SB, Sutton S, Burgess CC, Littlejohns P (1999) A Systematic Review of the Delay in Diagnosis/Treatment of Symptomatic Breast Cancer. NHS Cancer Research and Development Programme

[bib57] Worden JW, Weisman AD (1975) Psychosocial components of lagtime in cancer diagnosis. J Psychosom Res 19: 69–79 1142317 10.1016/0022-3999(75)90052-5

